# Supplementary figures and images for: Acetaminophen administration reduces acute kidney injury risk in critically ill patients with Clostridium difficile infection: A cohort study
Source: PLoS One. 2024 Dec 30;19(12):e0314902. doi: 10.1371/journal.pone.0314902 (PMC11684698; doi:10.1371/journal.pone.0314902)

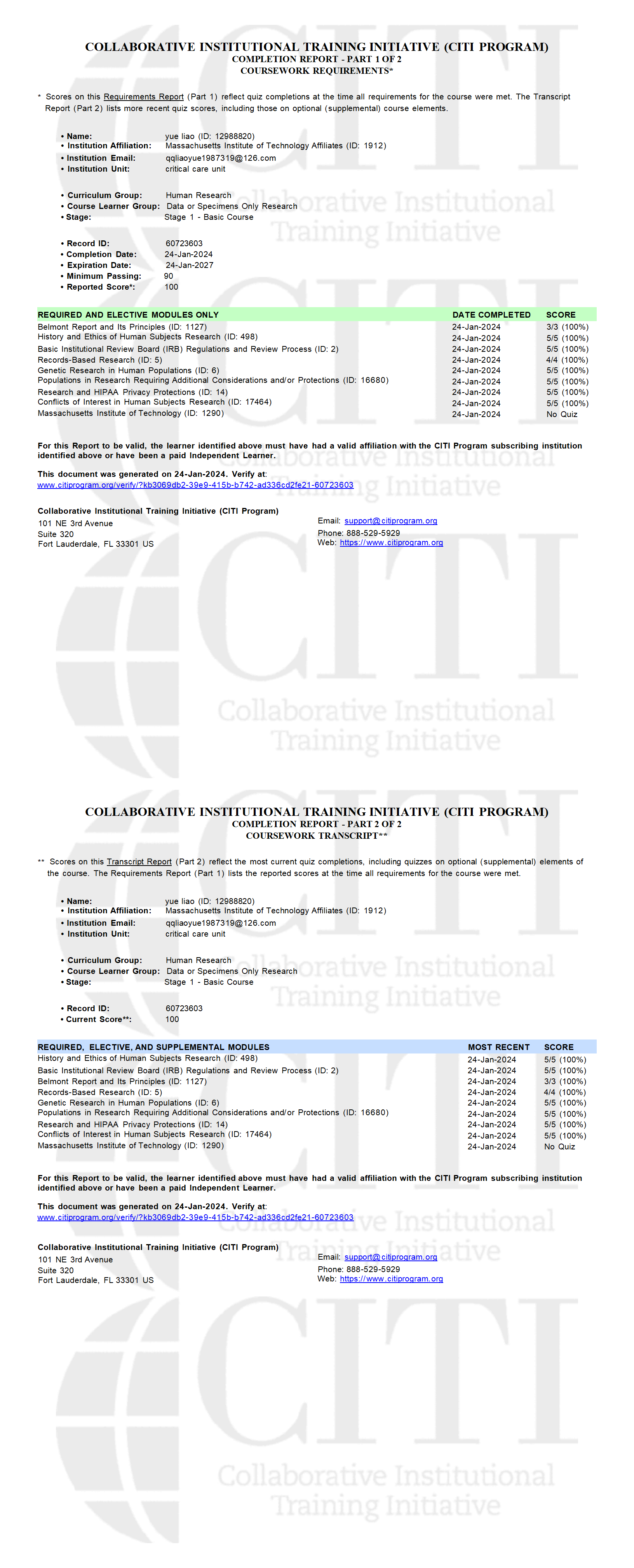

Supplement: S1 Fig — (TIF) [file pone.0314902.s001.tif]
