# Supplementary material for: Acetaminophen administration reduces acute kidney injury risk in critically ill patients with Clostridium difficile infection: A cohort study
Source: PLoS One. 2024 Dec 30;19(12):e0314902. doi: 10.1371/journal.pone.0314902 (PMC11684698; doi:10.1371/journal.pone.0314902)
Supplement: S1 Table — (DOCX) [file pone.0314902.s002.docx]

S1 Table | Multivariable logistic regression was used to assess the association between acetaminophen administration and the risk of AKI excluding patients with renal disease.

|  | N | Odds ratio of  acetaminophen used | 95% confidence interval | P-value |
| --- | --- | --- | --- | --- |
| Model 1 | 712 | 0.60 | (0.43~0.84) | 0.002 |
| Model 2 | 712 | 0.62 | (0.42~0.9) | 0.012 |

Adjusted covariates:

Model 1=acetaminophen administration.

Model 2=Model1+age+sex+heart rate+ dbp+ sbp+ temperature+ wbc+ platelets+ hemoglobin+ glucose+ bun+ creatinine+ potassium+ calcium+ SOFA score+ vasopressors use+ hypertension+ diabetes+ myocardial infarct+ congestive heart failure+ chronic pulmonary disease.
