# Supplementary material for: Acetaminophen administration reduces acute kidney injury risk in critically ill patients with Clostridium difficile infection: A cohort study
Source: PLoS One. 2024 Dec 30;19(12):e0314902. doi: 10.1371/journal.pone.0314902 (PMC11684698; doi:10.1371/journal.pone.0314902)
Supplement: Attachment — Submitted filename: Response to Reviewers.doc [file pone.0314902.s004.doc]

ID: PONE-D-24-23392

Title of Manuscript: Acetaminophen administration reduces acute kidney injury risk in critically ill patients with Clostridium difficile infection: a cohort study

Dear Editor Diana Laila Ramatillah, PhD and Reviewers：

On behalf of my co-authors, we greatly appreciate the careful review and comments from both you and the reviewers. We believe that by implementing the suggested changes, we now have a stronger manuscript entitled“Acetaminophen administration reduces acute kidney injury risk in critically ill patients with Clostridium difficile infection: a cohort study” (ID: PONE-D-24-23392) for submission to PLOS ONE. We look forward to your positive response to the revised work submitted here.

We present here point-to-point responses for each of the comments in the attached document“Response to Reviewers” and have revised our manuscript accordingly. We have updated the title to better reflect our study's findings:“Acetaminophen administration reduces acute kidney injury risk in critically ill patients with Clostridium difficile infection: a cohort study” (original title:The Relationship Between Acetaminophen Administration and Acute Kidney Injury in Critically Ill Patients with Clostridium difficile Infection: A Cohort Study)，which aligns with the primary outcome and study design as detailed in the manuscript. As suggested, We have further explored the mechanisms of acute kidney injury in patients with Clostridium difficile infection and how acetaminophen’s pharmacologic properties may counteract these processes in the introduction and discussion sections. Additionally, to ensure the robustness of our results, we conducted sensitivity analyses excluding patients with pre-existing kidney disease. These analyses confirmed the stability of our findings and are detailed in the supplementary materials, and we did not change the other statistics or results.We hope the revised manuscript could be acceptable for you.

There are no conflicts of interest regarding this work. All authors have read the revised manuscript and approved its submission to the PLOS ONE. Please do not hesitate to contact us if we can be of any further assistance.Thank you and best regards.

Yours Sincerely.

Xuewen Qiu

E-mail: qiuxuewen@cqu.edu.cn

Department of Pharmacy, Chongqing General Hospital, Chongqing University, Chongqing, China

**Reviewer #1:** I wish if the authors can briefly state the proposed possible mechanisms of action of acetaminophen in their manuscript, although the full detailed mechanisms are not fully understood according to the FDA and to try to explain the study results in the light of these mechanisms (e.g how the selectivity of acetaminophen towards COX-1, COX-2 and COX-3 variants can justify the observed results)

**Response：**Thank you for your valuable suggestions. We recognize the importance of detailing the mechanisms of action of acetaminophen in the paper to enhance the rationale of the study and the interpretation of the results. In the revised manuscript, we have added the following content to the introduction section**(Page 4, Line80-87):**

Acetaminophen serves as a standard antipyretic and analgesic agent in ICU [16,17]. It exerts its therapeutic effects by suppressing the cyclooxygenase activity of COX-1 and COX-2, thereby reducing the synthesis of prostaglandins (PGs). Additionally, acetaminophen inhibits other peroxidases, including myeloperoxidase, resulting in a decrease in the formation of halogenated oxidants, which may contribute to the slowing down of inflammation development[18]. Moreover, studies suggest that acetaminophen may protect kidney function, potentially by mitigating lipid peroxidation induced by acellular hemoglobin [19].

In the discussion section, we have added the following content**(Page 18, Line344-352):**

Investigations revealed that damage to the intestinal mucosa may precipitate the translocation of gut microbiota and its luminal contents into the systemic circulation, thereby leading to sepsis [12,33]. During sepsis, increased levels of CFHb may mediate lipid peroxidation, resulting in oxidative damage to renal tubules. Metabolites of this process, such as plasma F2-isoprostanes (F2-IsoPs), can further exacerbate kidney injury by inducing renal vasoconstriction [34]. Acetaminophen, attributed to its ferryl radical-neutralizing capacity, effectively inhibits hemoglobin-mediated lipid peroxidation. This property is linked to a significant decrease in early plasma F2-IsoPs levels, thereby alleviating the oxidative stress associated with septic conditions [20].

**Reviewer #2:**

1) . How does the dosage of acetaminophen affect the risk of acute kidney injury (AKI) in critically ill patients with Clostridium difficile infection (CDI) as observed in the study?

**Response：**Thank you for your valuable feedback. The impact of acetaminophen dosage on the risk of acute kidney injury (AKI) due to Clostridium difficile infection (CDI) in critically ill patients is indeed an important issue. We acknowledged this limitation in the original manuscript's limitations section. We attempted to extract dosage information for acetaminophen, but due to database constraints, we could not obtain specific dosage details for acetaminophen. Consequently, we were unable to assess the potential dose-response relationship. We have included this detail in the limitations of the manuscript, which can be found on**(Page19, Line373-376)：**

Third, the absence of detailed data on acetaminophen dosage precludes us from assessing a potential dose-response relationship, which is an area we aim to address in our ongoing research.

2) . What confounding factors were identified in the study, and how did adjusting for these factors influence the results regarding the association between acetaminophen dosage and AKI risk?

**Response：**Thank you for your insightful comments. In the multivariable analysis, confounding factor is an important issue,we performed some different statistical models to verify the results’ stability. Following are the details (1 or 2 or 3)[1,2]：

(1) Factors were chosen when adding it to this model and changed the matched odds ratio by at least 10 percent.

(2) For univariate analysis, we adjusted for variables, of which the p values were less than 0.1.

(3) For multivariable analysis, variables were chosen on the basis of previous findings and clinical constraints.

In our study, the adjustment models were as follows:

**TABLE 2**| Multivariable logistic regression to assess the association of acetaminophen administration and acute kidney injury using an extended model approach .

|  | **Odds ratio of**  **acetaminophen used** | **95% confidence interval** | **P-value** |
| --- | --- | --- | --- |
| Model 1 | 0.63 | (0.47~0.84) | 0.002 |
| Model 2 | 0.63 | (0.47~0.84) | 0.002 |
| Model 3 | 0.66 | (0.49~0.9) | 0.008 |
| Model 4 | 0.69 | (0.49~0.97) | 0.031 |
| Model 5 | 0.68 | (0.48~0.96) | 0.027 |

Model 1 = acetaminophen administration.

Model 2 = Model 1+ (age + sex).

Model 3 = Model 2 + (hypertension+ diabetes+ myocardial infarct+ congestive

heart failure+ chronic pulmonary disease+ renal disease).

Model 4 = Model 3+ (wbc+ heart rate+ dbp +temperature +glucose +bun+ creatinine+

potassium+ SOFA score+ vasopressors use).

Model 5 = Model 4 + (sbp +platelets +hemoglobin +calcium).

By incrementally adding these variables, we ensured that our findings regarding the association between acetaminophen usage and the prevention of AKI were robust and not attributable to any single confounding factor.

However, due to limitations in the database, we could not obtain specific dosage details for acetaminophen. We have included this specific information in the limitations of our manuscript, which can be found on **(Page 19, Line373-376):**

Third, the absence of detailed data on acetaminophen dosage precludes us from assessing a potential dose-response relationship, which is an area we aim to address in our ongoing research. We appreciate the opportunity to clarify our approach to addressing confounding factors and believe that these detailed adjustments strengthen the conclusions drawn from our study.

3) . What are the potential clinical implications of the study's findings on acetaminophen administration and AKI risk in patients with CDI, and how might these findings influence future practices in the management of critically ill patients?

**Response：**Thank you for your interest in our research, your suggestion is very valuable. Our study suggests that acetaminophen administration is associated with a reduced risk of AKI in patients with CDI. This could support the preferential use of acetaminophen over other antipyretics in this patient group. We've added a discussion on the potential clinical implications and future research directions in our conclusion to address these points **(Page 20, Line 382-387):**

“While these findings are promising, future research should include prospective, multicenter randomized controlled trials to definitively establish the causal relationship between early acetaminophen exposure and the prevention of AKI in CDI patients. These trials will be essential in guiding clinical decision-making and treatment guidelines for this patient population.”

1.	VanderWeele TJ. Principles of confounder selection. Eur J Epidemiol. 2019;34: 211–219. doi:10.1007/s10654-019-00494-6

2.	Greenland S. Invited commentary: variable selection versus shrinkage in the control of multiple confounders. Am J Epidemiol. 2008;167: 523–529; discussion 530-531. doi:10.1093/aje/kwm355

**Reviewer #3:** Dear Editor and Authors,

I would like to start by commending the authors on their important contribution to understanding the relationship between acetaminophen administration and the risk of acute kidney injury (AKI) in critically ill patients with Clostridioides difficile infection (CDI). The use of a large and well-validated dataset like MIMIC-IV adds strength to the findings, and the statistical methods used are robust and appropriate for the study design. The work is both timely and clinically relevant, given the ongoing challenge of AKI management in critically ill populations. I believe this study has the potential to impact clinical practice, and with some revisions, it will be even stronger.

Overall, this is a strong manuscript with valuable clinical insights. I hope the suggestions provided will help enhance its clarity and impact. Thank you again for your hard work and contribution to this important area of research.

I have provided detailed feedback on various sections of the manuscript to help enhance its clarity and scientific rigor.

Abstract

The abstract concisely summarizes the study but could benefit from some modifications for clarity:

* Background: The sentence "However, the correlation between its administration and acute kidney injury (AKI) among critically ill patients remains controversial" should be more specific. Adding the phrase “particularly in patients with Clostridioides difficile infection (CDI)” will provide clearer context earlier on.

**Response：**Thank you for your insightful suggestion, we agree that incorporating more detailed information will enhance the clarity of our narrative. In deference to your guidance, we have made the following modifications to the original text:

Background: However, the association between acetaminophen administration and acute kidney injury (AKI) among critically ill patients remains controversial, particularly lacking research in patients with Clostridioides difficile infection (CDI).

* Methods: The term "multivariate logistic regression" should be pluralized to "multivariate logistic regression models." Additionally, consider briefly mentioning how confounders were selected or adjusted for, to provide a more complete picture.

**Response：**Thank you for your constructive suggestions. We have implemented the following updates in our revised manuscript based on your suggestions:

We utilized multivariable logistic regression models adjusted for potential confounders based on statistical significance and clinical relevance, to investigate the association between acetaminophen exposure and the risk of AKI in patients with CDI. Additionally, subgroup analyses and sensitivity analysis were conducted to assess the robustness of our primary findings.

* Results: The phrase "we found that acetaminophen administration was associated with 32% lower risk" could be more smoothly expressed as "acetaminophen administration was associated with a 32% reduction in the risk of AKI." Furthermore, specifying "95% CI" instead of "CI" is more standard.

**Response：**We sincerely appreciated the valuable feedback. Based on your suggestion, we have revised the sentence for greater clarity and specified "95% CI" instead of "CI".

Results: After adjusting for potential confounders, acetaminophen administration was associated with a 32% reduction in the risk of AKI (OR = 0.68, 95%CI:0.48~0.96, P=0.027).

* Conclusion: To emphasize clinical relevance, you could rephrase the conclusion as: "Our study suggests that early acetaminophen administration may offer renal protection by reducing the risk of AKI in critically ill patients with CDI."

**Response：**Thank you for your constructive suggestion to enhance the clinical relevance of our conclusions. We have rephrased the conclusion to better emphasize the potential clinical implications of our study as per your recommendation:

Conclusion: Our study suggests that early acetaminophen administration may offer renal protection by reducing the risk of AKI in critically ill patients with CDI. Prospective, multicenter randomized controlled studies are needed to verify this finding.

Introduction

The introduction is well-written, but there are opportunities to strengthen the framing of the problem and the study's hypothesis:

* Clarity of Concepts: While the background on AKI and CDI is solid, the introduction could benefit from a more explicit explanation of the mechanism by which CDI might increase the risk of AKI. Discussing the toxins produced by Clostridioides difficile and how they exacerbate renal injury would help justify the study's focus.

**Response：**Thank you for your constructive suggestions. We have expanded the introduction to include a detailed discussion on the potential mechanisms by which CDI might increase the risk of AKI. Specifically, we have added a section that describes the toxins produced by Clostridioides difficile, and their potential role in exacerbating renal injury. This new section also discusses how these toxins contribute to inflammation, which could lead to AKI **(Page4, Line69-74)**:

CDI Patients exhibit an increased susceptibility to AKI, potentially attributable to diminished renal perfusion, oxidative stress, the effects of Clostridium difficile toxins, and inflammatory processes[7–11]. Toxins produced by Clostridium difficile damage intestinal wall cells, enter the circulation, and trigger a systemic inflammatory response [12,13]. The elevation of proinflammatory cytokines such as TNF-α and IFN-γ further causes multi-organ damage[14], which confers a significantly elevated mortality and are associated with progressive deterioration of renal function[7,15].

* Mechanistic Hypothesis: The potential mechanisms by which acetaminophen might protect against AKI (e.g., by reducing oxidative stress or inflammation) could be further elaborated. This would better support the rationale for the study and provide a clearer connection between the drug's properties and the expected outcomes.

**Response：**Thank you for your suggestion. We have expanded our discussion to include an overview of the potential mechanisms by which acetaminophen may protect against AKI **(Page4, Line80-87):**

Acetaminophen serves as a standard antipyretic and analgesic agent in ICU [16,17]. It exerts its therapeutic effects by suppressing the cyclooxygenase activity of COX-1 and COX-2, thereby reducing the synthesis of prostaglandins (PGs). Additionally, acetaminophen inhibits other peroxidases, including myeloperoxidase, resulting in a decrease in the formation of halogenated oxidants, which may contribute to the slowing down of inflammation development [18]. Moreover, studies suggest that acetaminophen may protect kidney function, potentially by mitigating lipid peroxidation induced by acellular hemoglobin [19].

* Literature Gap: While you highlight that prior studies have shown inconsistent results regarding acetaminophen and AKI, it would be beneficial to explore why these inconsistencies might exist (e.g., differences in patient populations, timing of administration, or methodological limitations of previous studies). This will strengthen the justification for your study.

**Response：**Thank you for your insightful comment. You are correct that prior studies have presented inconsistent findings regarding the relationship between acetaminophen and AKI. We have added a section in the discussion that explores potential reasons for these inconsistencies, and demonstrates the necessity of our study **(Page5, Line90-98):**

Previous studies have shown that acetaminophen administration was associated with reduced AKI[20–22], while others indicated a scarce association between them [23,24]. This variability can be attributed to differences in patient demographics, the timing of acetaminophen administration, and the methodological constraints across various research designs. Nonetheless, a consistent theme across these investigations is the ongoing research interest in the potential renal protective effects of acetaminophen. However, limited clinical studies have assessed the effect of acetaminophen exposure on AKI in CDI patients. Against this backdrop, this cohort study aims to explore the relationship between early acetaminophen administration and AKI among CDI patients in the ICU.

Methods

The methods section is detailed, but a few areas could use additional clarification or elaboration:

* Study Design and Data Source: It would be helpful to include more details about why MIMIC-IV was chosen as the data source for this study. Highlighting the database's strengths, such as its large size, diversity of patient populations, and detailed clinical variables, would justify its use.

**Response：**We appreciate your feedback and agree that providing more context on our choice of data source is important. In the revised manuscript, we have emphasized the large size and detailed clinical variables of the database in the methods section **(Page5, Line103-106):**

We enrolled patients with CDI from the MIMIC-IV (Medical Information Mart for Intensive Care IV, version 2.2) database of the Massachusetts Institute of Technology (MIT). MIMIC-IV contains detailed medical data from over 70,000 adult ICU admissions at Beth Israel Deaconess Medical Center in Boston between 2008 and 2019, including patient measurements, orders, diagnoses, procedures, and treatments [25]. Given that the information was sourced from publicly accessible databases, the need for informed consent was waived.

* Outcome Definitions: The use of KDIGO criteria to define AKI is appropriate, but a brief explanation of why this definition was selected (in comparison to other criteria) would provide additional context for readers who may not be as familiar with this classification.

**Response：**Thank you for your suggestion. We chose to define AKI using the KDIGO criteria because this standard has been widely accepted and adopted in the medical community. In the revised manuscript, we have provided a brief explanation of why this definition was chosen to provide additional context for readers. This explanation can be found in the first paragraph of “Definitions”section **(Page6, Line127-130):**

AKI was diagnosed and staged according to the Kidney Disease Improving Global Outcomes (KDIGO) criteria, which provide a standardized, evidence-based approach with superior diagnostic and prognostic accuracy, and broader acceptance in the global medical community compared to other classifications [27]. Consistent with these criteria, AKI was defined as an absolute increase in serum creatinine of ≥ 0.3 mg/dL within 48 h or≥ 1.5 times baseline within 7 days after admission date[28] .

* Covariates: The covariates are thoroughly described, but it would be helpful to explain the rationale for including specific variables like heart rate and blood pressure as potential confounders. This can clarify how you aimed to control for factors that may influence AKI risk.

**Response：**We appreciate your suggestion to provide further clarity on the selection of covariates in our analysis. Based on clinical experience and the results of relevant clinical studies (as shown in the table below), an increase in heart rate may be due to the body's response to pain, infection, or other stress factors, all of which can affect renal blood flow and function. Additionally, blood pressure is a critical determinant of renal perfusion. By adjusting for these variables, we aimed to control for their potential influence on the relationship between acetaminophen administration and the AKI risk.

| Journal | Title | First author | Year | Exposure | Outcome |
| --- | --- | --- | --- | --- | --- |
| Journal of internal medicine | Resting heart rate is associated with renal disease outcomes in patients with vascular disease: results of the ONTARGET and TRANSCEND studies | Schumacher | 2015 | Resting heart rate | Renal disease outcomes |
| American Journal of Nephrology | Renal function and cardiovascular response to mental stress | Seliger | 2008 | Cardiovascular response | Renal function |
| American Journal of Kidney Diseases | Pain and kidney function decline and mortality: a cohort study of US veterans | Ravel | 2016 | Pain | Kidney function |
| Critical Care | Blood pressure and acute kidney injury | Sato | 2017 | Blood pressure | Acute kidney injury |
| Kidney International | KDIGO 2021 clinical practice guideline for the management of blood pressure in chronic kidney disease | KDIGO Blood Pressure Work Group | 2021 | Guidelines that include optimal blood pressure targets for patients with chronic kidney disease to delay the deterioration of kidney function. | |

* Statistical Analysis: While the use of multiple imputation for handling missing data is appropriate, a brief justification for this method would be beneficial. Additionally, mention if any sensitivity analyses were conducted to assess the robustness of your findings in the face of imputed data.

**Response：**Thank you for your insightful comment. We elected to employ multiple imputation to address missing data, creating five complete datasets to reflect the uncertainty of the missing information. Each dataset was then analyzed, and the results were pooled to obtain the final estimates. This approach aims to reduce bias due to data absence and provides unbiased estimates of parameters for the complete dataset (Austin et al. 2021). Additionally, we conducted an analysis after excluding missing values to assess the consistency with the results obtained following imputation. The findings indicate that our results remain robust(Table 1). Furthermore, we conducted a sensitivity analysis after excluding patients with comorbid kidney disease. The findings showed that among the 712 patients without kidney disease, the use of acetaminophen was still significantly associated with a reduced risk of AKI( S1 Table). We have included the results of the sensitivity analysis in the supplementary materials：

Table 1 |Multivariable logistic regression was used to assess the association between acetaminophen administration and the risk of AKI excluding missing values.

|  | Odds ratio of  acetaminophen used | 95% confidence interval | P-value |
| --- | --- | --- | --- |
| Model 1 | 0.63 | (0.47~0.84) | 0.002 |
| Model 2 | 0.56 | (0.36~0.86) | 0.008 |

Adjusted covariates:

Model 1=acetaminophen administration.

Model 2=Model1+age+sex+heart rate+ dbp+ sbp+ temperature+ wbc+ platelets+ hemoglobin+

glucose+ bun+ creatinine+ potassium+ calcium+ SOFA score+ vasopressors use+ hypertension+

diabetes+ myocardial infarct+ congestive heart failure+ chronic pulmonary disease.

S1 Table |Multivariable logistic regression was used to assess the association between acetaminophen administration and the risk of AKI excluding patients with renal disease.

|  | N | Odds ratio of  acetaminophen used | 95% confidence interval | P-value |
| --- | --- | --- | --- | --- |
| Model 1 | 712 | 0.60 | (0.43~0.84) | 0.002 |
| Model 2 | 712 | 0.62 | (0.42~0.9) | 0.012 |

Adjusted covariates:

Model 1=acetaminophen administration.

Model 2=Model1+age+sex+heart rate+ dbp+ sbp+ temperature+ wbc+ platelets+ hemoglobin+

glucose+ bun+ creatinine+ potassium+ calcium+ SOFA score+ vasopressors use+ hypertension+

diabetes+ myocardial infarct+ congestive heart failure+ chronic pulmonary disease.

Austin, P. C., White, I. R., Lee, D. S., & van Buuren, S. (2021). Missing Data in Clinical Research: A Tutorial on Multiple Imputation. *The Canadian Journal of Cardiology*, *37*(9), 1322–1331. https://doi.org/10.1016/j.cjca.2020.11.010

Results

The results section is generally well-presented but could benefit from additional explanations and some restructuring:

* Clarity in Data Presentation: Although you refer to the tables for detailed results, it would be helpful to provide a more thorough narrative summary of key findings. For instance, instead of just directing readers to "See Table 1," briefly describe the most relevant characteristics, such as significant differences in age or baseline health between groups.

**Response：**Thank you for your valuable suggestions. Providing a narrative summary of key findings is essential for readers to understand our research. We have added detailed descriptions of the key results within the text. For instance, when discussing the baseline characteristics of the study population, we have provided the following narrative summary **(Page10, Line204-215):**

Baseline characteristics of the study population are listed in Table 1.The age of all participants was 66.8 ± 16.5 years, and 52.7% (519/984) were male. There were several significant differences in baseline health characteristics between the acetaminophen-exposed and non-exposed groups. Specifically, patients in the acetaminophen-exposed group had a higher average body temperature and a greater prevalence of dementia. Additionally, they exhibited lower arterial oxygen saturation (SPO2) levels. These patients were less likely to have comorbidities such as diabetes, renal disease, and chronic pulmonary disease, potentially reflecting differences in their overall health status. Regarding the development of AKI, we found a relatively high overall occurrence rate of 75.4% (742/984). The incidence of AKI for non-acetaminophen administration group and acetaminophen administration group was 79.8% (380/476) and 71.3% (362/508), respectively.

* Subgroup Analysis: The interaction effects from the subgroup analysis (Figure 2) are interesting but would benefit from further discussion. For example, why was there no significant interaction effect in certain subgroups? Offering potential explanations (e.g., sample size limitations, differences in baseline characteristics) would provide a more nuanced interpretation.

**Response：**Thank you for your comments on subgroup analysis results. We aimed to assess the consistency of our study's main findings across different populations through subgroup analysis. Our results show that there was no significant interaction effect between the use of acetaminophen and the risk of AKI in all tested subgroups, indicating that the results are robust across different populations. We agree that further discussion on potential reasons such as sample size and baseline differences is necessary when interaction effects are observed. However, in this study, due to the lack of significant interaction effects, we focused on discussing and interpreting our observed robust results, which enhances the credibility of our conclusions. We appreciate the opportunity to further clarify our findings.

* Visuals: The inclusion of a patient flow diagram (Figure 1) is helpful. Just ensure that it is clearly referenced in the text and consider briefly summarizing the patient selection process to help readers follow along more easily.

**Response：**Thank you for your suggestion. We have ensured that the patient flow diagram (Figure 1) is clearly referenced in the text and have briefly summarized the patient selection process within the text as well, so that readers will be able to more clearly understand our research methods and the criteria for patient inclusion **(Page9, Line191-197):**

From the MIMIC-IV 2.2 database, we included patients who were diagnosed with CDI and were admitted to the ICU for the first time (N = 1165). We excluded participants who were younger than 16 years or had a stay of less than 24 hours (N = 988). After excluding individuals without medical treatment data, the study ultimately resulted in a total of 984 patients for further analysis. Among them, 508 (51.63%) were acetaminophen users. A flowchart depicting the patient selection protocol for our study is shown in Fig 1.

Discussion

The discussion is thoughtful and connects the findings to existing literature, but it could be expanded in certain areas:

* Comparison with Previous Studies: While you effectively compare your findings with previous studies, it would be useful to delve deeper into why your results may differ from those of Patanwala et al. or Hiragi et al. For instance, were there differences in study design, patient populations, or AKI definitions that could explain the discrepancies? Offering potential reasons for these differences will show a critical engagement with the existing body of research.

**Response：**Thank you for your feedback. We acknowledge the differences in our study's results compared to those of Patanwala et al. and Hiragi et al. Upon reflection, we believe these discrepancies may be due to differences in sample size, variations in the study population, and changes in study design. We have added a brief discussion to the revised manuscript to address these potential reasons, and we believe this provides a clearer context for the research findings within the existing literature **(Page 17, Line318-322, Line324-328):**

Patanwala et al. conducted a retrospective cohort study suggesting that the use of acetaminophen may not reduce the occurrence or exacerbation of AKI in patients with severe sepsis (OR = 1.2; 95% CI = 0.6~2.4; P = 0.639) [23]. Compared with our study, their sample size was smaller and the study population was different, which may influence the comparability of the results. Furthermore, Patanwala et al. did not further assess the relationship between acetaminophen use and AKI in different subgroups. Hiragi et al. performed a self-controlled case series study indicating that the use of acetaminophen did not lead to the development and progression of AKI. However, their study design had significant differences compared to our cohort study, which may impact the interpretation of the findings. More importantly, they did not further explore whether acetaminophen exposure has a protective effect on renal function, which is a key focus of our research [24].

* Mechanistic Insights: A more detailed discussion of the potential mechanisms by which acetaminophen might reduce AKI would add depth to the paper. Consider expanding on the role of oxidative stress and lipid peroxidation in AKI and how acetaminophen’s pharmacologic properties may counteract these processes.

**Response：**Thank you for your valuable suggestion. We agree that a more detailed discussion of the potential mechanisms by which acetaminophen might reduce AKI would enhance the depth of our paper. We have expanded the discussion section to include its role in oxidative stress and lipid peroxidation in the pathogenesis of AKI **(Page18, Line342-352):**

Investigations revealed that damage to the intestinal mucosa can precipitate the translocation of gut microbiota and its luminal contents into the systemic circulation, thereby leading to sepsis[12,33]. During sepsis, increased levels of CFHb may mediate lipid peroxidation, resulting in oxidative damage to renal tubules. Metabolites of this process, such as plasma F2-isoprostanes (F2-IsoPs), can further exacerbate kidney injury by inducing renal vasoconstriction[34]. Acetaminophen, attributed to its ferryl radical-neutralizing capacity, effectively inhibits hemoglobin-mediated lipid peroxidation. This property is linked to a significant decrease in early plasma F2-IsoPs levels, thereby alleviating the oxidative stress associated with septic conditions [20].

* Limitations: You mention some limitations, but it would be beneficial to explicitly address additional potential weaknesses of the study. For example, since this is an observational study, residual confounding may still be present despite adjustments. Similarly, the use of retrospective data from MIMIC-IV could introduce bias related to selection or unmeasured variables. Discussing these limitations will add transparency and strengthen the validity of your findings.

**Response：**Thank you for your feedback. You're right that our observational study may have residual confounding despite adjustments, and the retrospective nature of the MIMIC-IV data could introduce bias. We've added a brief discussion on these limitations in the revised manuscript to enhance the transparency of our findings **(Page19, Line364-369):**

This study has several limitations. First, given its observational and non-randomized nature, we cannot fully exclude the possibility of residual confounding. Despite our comprehensive efforts to adjust for known confounders in our multivariable logistic regression models, the potential for unmeasured variables or selection bias, inherent in a retrospective cohort study，may still influence the results.

* Clinical Implications: Your findings have clear clinical implications, but they could be emphasized further. For instance, should acetaminophen now be recommended as a preventive measure for AKI in CDI patients? While this study provides evidence in favor of its use, you could mention the need for randomized controlled trials to definitively establish causality.

**Response：**We appreciate the opportunity to further clarify the implications of our study and the necessity for additional research. In response to your feedback, we have added a discussion on the need for randomized controlled trials to definitively establish a causal relationship **(Page 20, Line382-387):**

While these findings are promising, future research should include prospective, multicenter randomized controlled trials to definitively establish the causal relationship between early acetaminophen exposure and the prevention of AKI in CDI patients. These trials will be essential in guiding clinical decision-making and treatment guidelines for this patient population.

Additional Comments

* Formatting and Writing Style: Ensure that the formatting is consistent throughout, particularly in the use of citations and the presentation of numerical data. For instance, there are some minor issues like misplaced periods in "consequences1–4." or "infection3,5" that should be corrected for consistency.

**Response：**Thank you for pointing out the formatting and writing style issues in our manuscript. We have corrected the formatting issues, including the misplaced periods in citations, to ensure consistency throughout the manuscript. The revised document now adheres to the journal's formatting standards.We appreciate your attention to detail and have taken the necessary steps to enhance the manuscript's overall quality and presentation.

* Conclusion: Both the abstract and main text conclusions could be strengthened by suggesting future research directions. For example, while acetaminophen shows promise in reducing AKI risk, a call for randomized controlled trials would make the conclusion more forward-looking.

**Response：**We appreciate your guidance in helping us make our conclusions more forward-looking. We have revised the conclusions in the abstract and the main text to include a call for future research.

Conclusion in the abstract: Our study suggests that early acetaminophen administration may offer renal protection by reducing the risk of AKI in critically ill patients with CDI. Prospective, multicenter randomized controlled studies are needed to verify this finding.

Conclusion in the main text: In summary, we provide the first evidence that the administration of acetaminophen within the first day of ICU admission for patients with CDI can reduce the incidence of AKI, and the protective effect of acetaminophen remains significant for the risk of severe AKI. While these findings are promising, future research should include prospective, multicenter randomized controlled trials to definitively establish the causal relationship between early acetaminophen exposure and the prevention of AKI in CDI patients. These trials will be essential in guiding clinical decision-making and treatment guidelines for this patient population.

**Reviewer #4:** Thank you for the opportunity to review the manuscript titled, "The Relationship Between Acetaminophen Administration and Acute Kidney Injury in Critically Ill Patients with Clostridium difficile Infection: A Cohort Study". The manuscript presents a comprehensive analysis of the relationship between early acetaminophen administration and acute kidney injury (AKI) in critically ill patients with Clostridium difficile infection (CDI) admitted into intensive care unit (ICU). While the study addresses a relevant clinical research problem and has strong potential to improve clinical practice, there are some issues and recommendations the authors needs to address to enhance the robustness and quality of their study.

The following are my comments describing the issues and recommendations:

Comments:

1. Title page

I. The corresponding author should provide an ORCID iD in line with PLOS submission guidelines

**Response：**We're grateful for your reminder. We have added the corresponding author's ORCID ID to the manuscript as per PLOS guidelines（0009-0006-1303-3655）.

2. Introduction

I. The authors cited previous studies with conflicting results but did not provide a thorough analysis of why these discrepancies exist or how their study aims to resolve them. They should provide additional literature review on the mechanism by which acetaminophen is hypothesized to have a protective effect on the kidneys in CDI patients. This would further strengthen the rationale for this study.

**Response：**Thank you for your feedback. We have expanded our discussion to include a more detailed examination of the potential reasons for the conflicting results found in previous studies **(Page5, Line90-98):**

Previous studies have shown that acetaminophen administration was associated with reduced AKI[20–22], while others indicated a scarce association between them [23,24]. This variability can be attributed to differences in patient demographics, the timing of acetaminophen administration, and the methodological constraints across various research designs. Nonetheless, a consistent theme across these investigations is the ongoing research interest in the potential renal protective effects of acetaminophen. However, limited clinical studies have assessed the effect of acetaminophen exposure on AKI in CDI patients. Against this backdrop, this cohort study aims to explore the relationship between early acetaminophen administration and AKI among CDI patients in the ICU.

Additionally, we have also elaborated on the potential mechanisms by which acetaminophen may exert a protective effect against AKI, particularly focusing on its role in reducing oxidative stress and inflammation **(Page4, Line80-87):**

Acetaminophen serves as a standard antipyretic and analgesic agent in ICU [16,17]. It exerts its therapeutic effects by suppressing the cyclooxygenase activity of COX-1 and COX-2, thereby reducing the synthesis of prostaglandins (PGs). Additionally, acetaminophen inhibits other peroxidases, including myeloperoxidase, resulting in a decrease in the formation of halogenated oxidants, which may contribute to the slowing down of inflammation development [18]. Moreover, studies suggest that acetaminophen may protect kidney function, potentially by mitigating lipid peroxidation induced by acellular hemoglobin [19].

3. Material and methods: The study adheres to general scientific principles, but some areas require more clarity and justification.

I. The definition of acetaminophen exposure as administration within the first day of ICU admission is a somewhat narrow definition, as longer durations of exposure might influence outcomes. The authors should clarify the rationale behind this cutoff and why those who were commenced after 24 hours of ICU admission were excluded

**Response：**Thank you for your insightful comment. Our initial aim was to assess the impact of early acetaminophen administration on the risk of acute kidney injury (AKI) in patients with CDI. A randomized, placebo-controlled trial revealed that the use of acetaminophen within 24 hours of ICU admission in critically ill adult patients may mitigate oxidative damage and improve kidney function(Janz et al. 2015). To ensure the accuracy and reliability of our results, we initially included only those patients who received acetaminophen within the first 24 hours of ICU admission.

Your suggestion has prompted us to reconsider our definition of exposure duration. Based on your feedback, we have expanded our analysis to include patients who used acetaminophen within 48 hours after ICU admission. Our preliminary analysis showed that within this extended time window, 617 patients used acetaminophen, and 367 did not (Table 1).

Given the difference in sample size between the two groups, we further conducted propensity score matching (PSM), resulting in 239 patients in both the exposed and unexposed groups

(Table 2).

Our results showed that the risk of AKI was significantly reduced in patients who used acetaminophen within 48 hours after ICU admission, both before and after PSM：

Table 1 | Multivariable logistic regression was used to assess the association between acetaminophen administration and the risk of AKI (before PSM).

|  | Odds ratio of  acetaminophen used | 95% confidence interval | P-value |
| --- | --- | --- | --- |
| Model 1 | 0.64 | (0.47~0.88) | 0.005 |
| Model 2 | 0.62 | (0.39~0.98) | 0.041 |

Adjusted covariates:

Model 1=acetaminophen administration.

Model 2=Model1+age+sex+heart rate+ dbp+ sbp+ temperature+ wbc+ platelets+ hemoglobin+ glucose+ bun+ creatinine+ potassium+ calcium+ SOFA score+ vasopressors use+ hypertension+ diabetes+ myocardial infarct+ congestive heart failure+ chronic pulmonary disease+

renal disease.

Table 2 | Multivariable logistic regression was used to assess the association between acetaminophen administration and the risk of AKI (after PSM).

|  | Odds ratio of  acetaminophen used | 95% confidence interval | P-value |
| --- | --- | --- | --- |
| Model 1 | 0.72 | (0.44~1.16) | 0.177 |
| Model 2 | 0.63 | (0.41~0.97) | 0.037 |

Adjusted covariates:

Model 1=acetaminophen administration.

Model 2=Model1+age+sex+heart rate+ dbp+ sbp+ temperature+ wbc+ platelets+ hemoglobin+ glucose+ bun+ creatinine+ potassium+ calcium+ SOFA score+ vasopressors use+ hypertension+ diabetes+ myocardial infarct+ congestive heart failure+ chronic pulmonary disease+

renal disease.

We appreciate your feedback and have taken the opportunity to clarify our methods and their implications for the study's results.

Janz, D. R., Bastarache, J. A., Rice, T. W., Bernard, G. R., Warren, M. A., Wickersham, N., et al. (2015). Randomized, placebo-controlled trial of acetaminophen for the reduction of oxidative injury in severe sepsis: the Acetaminophen for the Reduction of Oxidative Injury in Severe Sepsis trial*. Critical Care Medici*ne*,* 43(3), 534–541. https://doi.org/10.1097/CCM.0000000000000718

II. The authors did not provide information regarding the use of opiods and nonsteroidal anti-imflammatory, which are associated with an increased risk of AKI in the study population. This should be included as part of limitations of this study if the data cannot be extracted from the MIMIC IV version 2.2 database.

**Response：**Thank you for your valuable suggestions. We had previously extracted drugs with the potential for nephrotoxicity and adjusted for them, but found that they did not affect the results, hence they were not included in the final output. Following your advice, we extracted data on patient use of opioids and NSAIDs (Table 1) from the database and conducted an analysis. Our results indicate that although the use of these medications may impact the risk of AKI, the significant association between acetaminophen use and the reduced risk of AKI remains after considering these potential confounding factors (Table 2):

Table 1 | The list of high-risk nephrotoxic medications includes opioids and NSAIDs.

| Indometacin | Ibuprofen | Meloxicam | Naproxen |
| --- | --- | --- | --- |
| Morphine | Fentanyl | Pethidine | Codeine |

Table 2 | Multivariable logistic regression to assess the association of acetaminophen administration with risk of AKI （with adjustments of nephrotoxic medications use）.

|  | **N** | **Odds ratio of**  **acetaminophen used** | **95% confidence interval** | **P-value** |
| --- | --- | --- | --- | --- |
| Model 1 | 984 | 0.63 | (0.47~0.84) | 0.002 |
| Model 2 | 984 | 0.68 | (0.48~0.96) | 0.027 |

Adjusted covariates:

Model 1=acetaminophen administration.

Model 2 = Model 1+age+sex+heart rate+dbp+sbp+temperature+wbc+platelets+hemoglobin+glucose+bun+creatinine+potassium+calcium+SOFA score+ vasopressors use+ nephrotoxic medications use+ hypertension+ diabetes+ myocardial infarct +congestive heart failure+ chronic

pulmonary disease+ renal disease.

III. They should explain why adverse events of acetaminophen administration such hypersensitivity reactions, acute liver failure, etc were not investigated as one of the secondary outcome measures?

**Response：**Thank you for your suggestion. We could not extract the data on hypersensitivity reactions due to constraints within the database. While we did manage to extract data on acute liver failure using ICD codes K720, K7200, and K7201, the sample size was too small, with only 19 cases identified. Given this limited sample size, we did not include it as a secondary outcome in our analysis.

4. Data analysis

The statistical analysis is generally sound and adheres to standard practices for cohort studies.

I. Although the subgroup analyses of the covariates including comorbid conditions were done , the interaction terms are not well-explained, and the justification for specific subgroups analysis is also not clear. The authors need to provide more rationale for the subgroups used in the analysis.

**Response：**Thank you for your comments on subgroup analysis results. We aimed to assess the consistency of our study's main findings across different populations through subgroup analysis. Our results show that there was no significant interaction effect between the use of acetaminophen and the risk of AKI in all tested subgroups, indicating that the results are robust across different populations. We agree that further discussion on potential reasons such as sample size and baseline differences is necessary when interaction effects are observed. However, in this study, due to the lack of significant interaction effects, we focused on discussing and interpreting our observed robust results, which enhances the credibility of our conclusions. We appreciate the opportunity to further clarify our findings.

II. Excluding patients with renal diseases would have simplified the analysis by reducing the need to adjust for this significant confounder. The effects of acetaminophen would have been clearer in patient without pre-existing kidney problems.

**Response：**Thank you for your suggestion. We agree that the potential effects of acetaminophen might be clearer in patients without pre-existing kidney problems. To address this concern, we conducted an additional sensitivity analysis excluding patients with comorbid kidney disease. The results showed that among the 712 patients without kidney disease, the use of acetaminophen was still significantly associated with a reduced risk of AKI. We have included the results of the sensitivity analysis in the supplementary materials:

S1 Table | Multivariable logistic regression was used to assess the association between acetaminophen administration and the risk of AKI excluding patients with renal disease.

|  | N | Odds ratio of  acetaminophen used | 95% confidence interval | P-value |
| --- | --- | --- | --- | --- |
| Model 1 | 712 | 0.60 | (0.43~0.84) | 0.002 |
| Model 2 | 712 | 0.62 | (0.42~0.9) | 0.012 |

Adjusted covariates:

Model 1=acetaminophen administration.

Model 2=Model1+age+sex+heart rate+ dbp+ sbp+ temperature+ wbc+ platelets+ hemoglobin+ glucose+ bun+ creatinine+ potassium+ calcium+ SOFA score+ vasopressors use+ hypertension+ diabetes+ myocardial infarct+ congestive heart failure+ chronic pulmonary disease.

II. There is a heavy reliance on odds ratios, but a computation of the absolute risk differences would help to contextualize the findings of a decrease in incidence of AKI by 32%.

**Response：**Thank you for your insightful suggestion. We have calculated the absolute risk difference (ARD) to provide additional context for the findings. We believe this additional analysis enhances the interpretability of our results and aligns with the best practices in presenting risk estimates in medical research. In the abstract of the revised manuscript, we have added **(Page2-3, Line44-46):**

Compared to the non-acetaminophen administration group, the risk of AKI was lower in the acetaminophen administration group (absolute risk difference: -8.5%, 95% CI: -13.83% to -3.17%, P < 0.01). After adjusting for potential confounders, acetaminophen administration was associated with a 32% reduction in the risk of AKI (OR = 0.68, 95% CI: 0.48 to 0.96, P = 0.027).

In the results section of the revised manuscript, we have added **(Page12, Line226-229):**

The acetaminophen administration group exhibited a lower risk of AKI compared to the non-acetaminophen administration group, with an absolute risk difference of -8.5% (95% CI: -13.83% to -3.17%, P < 0.01). This significant reduction in AKI risk associated with acetaminophen administration was observed in both univariable and multivariable logistic regression models.

IV. In addition to the logistic regression analysis, the use of cox regression and hazard ratio analysis would have enhanced the robutness of the study findings, especially by providing insights into the timing of AKI onset relative to acetaminophen administration.

**Response：** Thank you for your valuable feedback. We agree that Cox regression and hazard ratios would provide valuable insights into the timing of AKI onset relative to acetaminophen administration, our dataset only includes binary AKI outcome data without specific onset times. Therefore, we were unable to apply a Cox regression model. Given this limitation, we conducted a logistic regression analysis to assess the relationship between acetaminophen dosage and AKI risk, adjusting for potential confounders. We appreciate your suggestion and will consider incorporating time-dependent analysis in future studies when more detailed data are available.

5. Results:

I. The unit of measurement for age in this statement ''The age of all participants was 66.8 ± 16.5" was not stated.

**Response：**Thank you for pointing out the missing unit of measurement. We have added "years" in the revised manuscript to clarify the age unit. The corrected content now reads: "The age of all participants was 66.8 ± 16.5 years."

III. Each table should be placed in the manuscript file directly after the paragraph in which it was first cited.

**Response：**Thank you for your guidance. We have adjusted the placement of each table to follow directly after the paragraph where it is first cited, ensuring consistency throughout the manuscript.

6. Reference

I. The authors did not comply with PLOS recommendation of using Vancouver reference style as outlined by the International Committee of Medical Journal Editors (ICMJE).

**Response：**Thank you for your feedback. We have now updated all references to comply with the Vancouver style, ensuring consistency with the expectations of PLOS.

7. Writing

I. The manuscript did not include page and line numbers in accordance with PLOS submission guidelines.

**Response：**Thank you for your feedback. We have added page and line numbers to our manuscript as per PLOS submission guidelines. This will ensure that the formatting is consistent throughout the document and will aid in the review process. We appreciate your guidance on this matter.

II. Some typographical and grammatical errors need to be addressed: For eamples 'our aim is to' was use instead of 'our aim was to" in the abstract, adults was spelt as adluts in the result section, "After adjusted for potential confounders" should written as " After adjusting for confounders" in the abstracts. The appropriate use of indefinite and definite articles through out the manuscript.

**Response：**Thank you for bringing these errors to our attention. We have carefully reviewed the manuscript and made the following corrections:

1.Changed "our aim is to" to "our aim was to" in the abstract.

2.Corrected the misspelling of "adults" to the correct form in the results section.

3.Amended "After adjusted for potential confounders" to "After adjusting for confounders" in the abstract.

4.Ensured the appropriate use of indefinite and definite articles throughout the manuscript.

We appreciate your diligence in helping us improve the quality and presentation of our paper.

II. The were numerous use of single spacing between words and

**Response：**Thank you for bringing this to our attention. We have corrected the spacing errors throughout the manuscript.
